# (*E*)-3,4-Dihydroxy­benzaldehyde 4-methyl­thio­semicarbazone

**DOI:** 10.1107/S1600536808034326

**Published:** 2008-10-25

**Authors:** Yang Farina, Jim Simpson

**Affiliations:** a School of Chemical Sciences and Food Technology, Faculty of Science and Technology, Universiti Kebangsaan Malaysia, 43600 UKM Bangi, Selangor, Malaysia; b Department of Chemistry, University of Otago, PO Box 56, Dunedin, New Zealand

## Abstract

The title compound, C_9_H_11_N_3_O_2_S, adopts an *E* configuration with respect to the C=N bond. The mol­ecule is approximately planar, with an r.m.s. deviation from the mean plane through all 15 non-H atoms of 0.152 Å; the dihedral angle between the benzene ring plane and the least-squares plane through the thio­semicarbazone unit is 12.48 (7)°. A weak intra­molecular N—H⋯N inter­action contributes to the planarity of the semicarbazone unit. Centrosymmetric pairs of O—H⋯O and N—H⋯S hydrogen bonds form chains along *c*, generating *R*_2_^2^(10) and *R*_2_^2^(8) ring motifs, respectively. In the crystal structure, these chains are further linked by inter­molecular O—H⋯S and C—H⋯O inter­actions, forming stacks down the *c* axis.

## Related literature

For the biological activity of thio­semicarbazones, see: de Sousa *et al.* (2007 ▶). For related structures, see: Kayed *et al.* (2008 ▶); Tan *et al.* (2008*a* ▶,*b* ▶). For hydrogen-bonding patterns, see: Bernstein *et al.* (1995 ▶). For reference structural data, see: Allen *et al.* (1987 ▶).
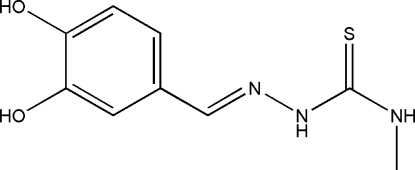


## Experimental

### 

#### Crystal data

C_9_H_11_N_3_O_2_S
*M* *_r_* = 225.27
Monoclinic, 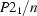

*a* = 6.8502 (9) Å
*b* = 14.911 (2) Å
*c* = 10.6299 (13) Å
β = 107.894 (6)°
*V* = 1033.3 (2) Å^3^
*Z* = 4
Mo *K*α radiation
μ = 0.30 mm^−1^
*T* = 92 (2) K
0.23 × 0.15 × 0.13 mm

#### Data collection

Bruker APEXII CCD area-detector diffractometer
Absorption correction: multi-scan (*SADABS*; Bruker, 2006 ▶) *T*_min_ = 0.799, *T*_max_ = 0.962
17952 measured reflections
3696 independent reflections
2974 reflections with *I* > 2σ(*I*)
*R*_int_ = 0.044

#### Refinement

*R*[*F* ^2^ > 2σ(*F* ^2^)] = 0.045
*wR*(*F* ^2^) = 0.112
*S* = 1.07
3696 reflections
149 parameters
4 restraints
H atoms treated by a mixture of independent and constrained refinement
Δρ_max_ = 0.49 e Å^−3^
Δρ_min_ = −0.32 e Å^−3^

### 

Data collection: *APEX2* (Bruker, 2006 ▶); cell refinement: *APEX2* and *SAINT* (Bruker 2006 ▶); data reduction: *SAINT*; program(s) used to solve structure: *SHELXS97* (Sheldrick, 2008 ▶); program(s) used to refine structure: *SHELXL97* (Sheldrick, 2008 ▶) and *TITAN* (Hunter & Simpson, 1999 ▶); molecular graphics: *SHELXTL* (Sheldrick, 2008 ▶) and *Mercury* (Macrae *et al.*, 2006 ▶); software used to prepare material for publication: *SHELXL97*, *enCIFer* (Allen *et al.*, 2004 ▶), *PLATON* (Spek, 2003 ▶) and *publCIF* (Westrip, 2008 ▶).

## Supplementary Material

Crystal structure: contains datablocks global, I. DOI: 10.1107/S1600536808034326/pv2112sup1.cif

Structure factors: contains datablocks I. DOI: 10.1107/S1600536808034326/pv2112Isup2.hkl

Additional supplementary materials:  crystallographic information; 3D view; checkCIF report

## Figures and Tables

**Table 1:** Hydrogen-bond geometry (Å, °)

*D*—H⋯*A*	*D*—H	H⋯*A*	*D*⋯*A*	*D*—H⋯*A*
N3—H3*N*⋯N1	0.879 (9)	2.220 (18)	2.6282 (17)	108.0 (14)
O3—H3⋯O4^i^	0.837 (9)	2.070 (14)	2.8071 (14)	146.5 (19)
O4—H4⋯S1^ii^	0.836 (9)	2.369 (10)	3.1899 (11)	167.1 (19)
N2—H2*N*⋯S1^iii^	0.876 (9)	2.785 (12)	3.5766 (13)	150.9 (15)
C9—H9*A*⋯O4^iv^	0.98	2.56	3.435 (2)	148

Symmetry codes: (i) 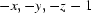; (ii) 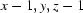; (iii) 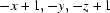; (iv) 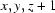.

**Fig. 1.**
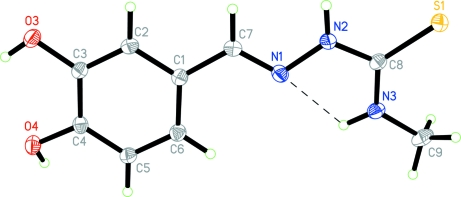
The structure of (I), with atom labels and 50% probability displacement ellipsoids for non-H atoms. An intramolecular hydrogen bond is drawn as a dashed line.

**Fig. 2.**
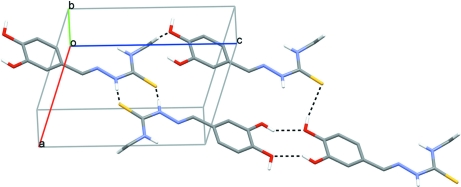
Crystal packing of (I) viewed down the c axis with hydrogen bonds drawn as dashed lines. H-atoms not involved in H-bonding have been excluded.

## supplementary crystallographic information

### Comment

Thiosemicarbazones are a class of compounds that have been extensively investigated because of their biological activity (de Sousa *et al.*, 2007). As a continuation of our work on thiosemicarbazone compounds as potential ligands in transition metal chemistry (Kayed *et al.*, 2008; Tan *et al.*, 2008*a*,*b*) we report here the structure of the title compound, (I).

The molecule of (I) (Fig. 1) is approximately planar with a dihedral angle of 12.48 (7)° between the phenyl ring plane and the least squares plane through the C7/N1/N2/C8/S2/N3 thiosemicarbazone moiety. The planarity of this section of the molecule is aided by a weak intramolecular N3—H3N···N1 interaction. The molecule adopts an *E* configuration with respect to the C═N bond and bond distances are normal (Allen *et al.*, 1987).

Pairs of O3—H3···O4 hydrogen bonds generate a centrosymmetric *R*^2^_2_(10) ring motif (Bernstein *et al.*, 1995) and, together with the *R*^2^_2_(8) ring generated by N2—H2N···S1 interactions, form an unusual molecular trimer. The crystal structure is further stabilized by O4—H4···S1 and C9—H9A···O4 contacts, Table 1, Fig. 2.

### Experimental

A 1:1 mixture of 3,4-dihydroxybenzaldehyde and N-methylhydrazinecarbothioamide was heated under reflux in ethanol for 2 hours. The solid product which separated upon cooling was filtered and recrystallised from methanol to afford colourless blocks of (I) in 54% yield (m.p. 418-419 K).

### Refinement

The H atoms bound to N and O atoms were located in a difference electron density map and refined freely with *U*_iso_ = 1.2*U*_eq_ (N) and *U*_iso_ = 1.5*U*_eq_ (O). All other H-atoms were refined using a riding model with d(C—H) = 0.95 Å, *U*_iso_= 1.2*U*_eq_ (C) for aryl and 0.98 Å, *U*_iso_ = 1.5*U*_eq_ (C) for methyl H atoms.

### Crystal data

**Table:**

C_9_H_11_N_3_O_2_S	*F*(000) = 472
*M**_r_* = 225.27	*D*_x_ = 1.448 Mg m^−^^3^
Monoclinic, *P*2_1_/*n*	Mo *K*α radiation, λ = 0.71073 Å
Hall symbol: -P 2yn	Cell parameters from 3847 reflections
*a* = 6.8502 (9) Å	θ = 2.4–29.8°
*b* = 14.911 (2) Å	µ = 0.30 mm^−^^1^
*c* = 10.6299 (13) Å	*T* = 92 K
β = 107.894 (6)°	Plate, colourless
*V* = 1033.3 (2) Å^3^	0.23 × 0.15 × 0.13 mm
*Z* = 4	

### Data collection

**Table:**

Bruker APEXII CCD area-detector diffractometer	3696 independent reflections
Radiation source: fine-focus sealed tube	2974 reflections with *I* > 2σ(*I*)
graphite	*R*_int_ = 0.044
ω scans	θ_max_ = 33.0°, θ_min_ = 3.4°
Absorption correction: multi-scan (SADABS; Bruker, 2006)	*h* = −10→10
*T*_min_ = 0.799, *T*_max_ = 0.962	*k* = −22→18
17952 measured reflections	*l* = −16→15

### Refinement

**Table:**

Refinement on *F*^2^	Primary atom site location: structure-invariant direct methods
Least-squares matrix: full	Secondary atom site location: difference Fourier map
*R*[*F*^2^ > 2σ(*F*^2^)] = 0.045	Hydrogen site location: inferred from neighbouring sites
*wR*(*F*^2^) = 0.112	H atoms treated by a mixture of independent and constrained refinement
*S* = 1.07	*w* = 1/[σ^2^(*F*_o_^2^) + (0.0412*P*)^2^ + 0.5756*P*] where *P* = (*F*_o_^2^ + 2*F*_c_^2^)/3
3696 reflections	(Δ/σ)_max_ < 0.001
149 parameters	Δρ_max_ = 0.49 e Å^−^^3^
4 restraints	Δρ_min_ = −0.32 e Å^−^^3^

### Special details

**Table:**

Geometry. All e.s.d.'s (except the e.s.d. in the dihedral angle between two l.s. planes) are estimated using the full covariance matrix. The cell e.s.d.'s are taken into account individually in the estimation of e.s.d.'s in distances, angles and torsion angles; correlations between e.s.d.'s in cell parameters are only used when they are defined by crystal symmetry. An approximate (isotropic) treatment of cell e.s.d.'s is used for estimating e.s.d.'s involving l.s. planes.
Refinement. Refinement of *F*^2^ against ALL reflections. The weighted *R*-factor *wR* and goodness of fit *S* are based on *F*^2^, conventional *R*-factors *R* are based on *F*, with *F* set to zero for negative *F*^2^. The threshold expression of *F*^2^ > σ(*F*^2^) is used only for calculating *R*-factors(gt) *etc*. and is not relevant to the choice of reflections for refinement. *R*-factors based on *F*^2^ are statistically about twice as large as those based on *F*, and *R*- factors based on ALL data will be even larger.

### Fractional atomic coordinates and isotropic or equivalent isotropic displacement parameters (Å^2^)

**Table:**

	*x*	*y*	*z*	*U*_iso_*/*U*_eq_	
C1	0.2002 (2)	0.06919 (9)	−0.01216 (13)	0.0157 (2)	
C2	0.2338 (2)	0.00229 (9)	−0.09567 (13)	0.0165 (2)	
H2	0.3237	−0.0460	−0.0590	0.020*	
C3	0.1378 (2)	0.00557 (9)	−0.23119 (13)	0.0160 (2)	
O3	0.17512 (16)	−0.06151 (7)	−0.30786 (10)	0.0213 (2)	
H3	0.110 (3)	−0.0532 (14)	−0.3876 (10)	0.032*	
C4	0.0039 (2)	0.07616 (9)	−0.28381 (13)	0.0166 (2)	
O4	−0.09897 (16)	0.07447 (7)	−0.41779 (10)	0.0206 (2)	
H4	−0.2160 (18)	0.0940 (13)	−0.424 (2)	0.031*	
C5	−0.0247 (2)	0.14471 (9)	−0.20207 (13)	0.0183 (3)	
H5	−0.1111	0.1940	−0.2392	0.022*	
C6	0.0727 (2)	0.14133 (10)	−0.06654 (14)	0.0180 (3)	
H6	0.0523	0.1881	−0.0111	0.022*	
C7	0.2988 (2)	0.05936 (9)	0.13036 (13)	0.0168 (2)	
H7	0.3900	0.0107	0.1623	0.020*	
N1	0.26441 (18)	0.11527 (8)	0.21297 (11)	0.0174 (2)	
N2	0.36894 (18)	0.09854 (8)	0.34422 (11)	0.0182 (2)	
H2N	0.456 (2)	0.0539 (9)	0.3669 (17)	0.022*	
C8	0.3229 (2)	0.14654 (9)	0.43924 (13)	0.0163 (2)	
S1	0.46023 (5)	0.12889 (2)	0.60055 (3)	0.01943 (10)	
N3	0.17327 (18)	0.20621 (8)	0.39921 (12)	0.0188 (2)	
H3N	0.107 (3)	0.2075 (12)	0.3142 (10)	0.023*	
C9	0.1019 (2)	0.26414 (10)	0.48622 (16)	0.0259 (3)	
H9A	0.0204	0.2289	0.5298	0.039*	
H9B	0.0171	0.3123	0.4343	0.039*	
H9C	0.2202	0.2902	0.5532	0.039*	

### Atomic displacement parameters (Å^2^)

**Table:**

	*U*^11^	*U*^22^	*U*^33^	*U*^12^	*U*^13^	*U*^23^
C1	0.0148 (5)	0.0165 (6)	0.0166 (5)	−0.0009 (5)	0.0061 (4)	−0.0004 (5)
C2	0.0158 (6)	0.0143 (6)	0.0202 (6)	0.0002 (5)	0.0067 (5)	0.0006 (5)
C3	0.0160 (6)	0.0148 (6)	0.0182 (6)	−0.0020 (5)	0.0067 (5)	−0.0019 (5)
O3	0.0239 (5)	0.0197 (5)	0.0194 (5)	0.0032 (4)	0.0055 (4)	−0.0040 (4)
C4	0.0149 (5)	0.0199 (6)	0.0153 (5)	−0.0006 (5)	0.0052 (4)	−0.0007 (5)
O4	0.0190 (5)	0.0258 (5)	0.0164 (4)	0.0032 (4)	0.0044 (4)	−0.0027 (4)
C5	0.0189 (6)	0.0172 (6)	0.0184 (6)	0.0031 (5)	0.0052 (5)	0.0000 (5)
C6	0.0172 (6)	0.0188 (6)	0.0182 (6)	0.0013 (5)	0.0058 (5)	−0.0009 (5)
C7	0.0157 (6)	0.0158 (6)	0.0187 (6)	0.0002 (5)	0.0050 (5)	0.0016 (5)
N1	0.0163 (5)	0.0198 (6)	0.0152 (5)	0.0012 (4)	0.0037 (4)	0.0011 (4)
N2	0.0200 (5)	0.0181 (6)	0.0160 (5)	0.0049 (4)	0.0050 (4)	0.0005 (4)
C8	0.0165 (6)	0.0141 (6)	0.0193 (6)	−0.0018 (5)	0.0072 (5)	−0.0010 (5)
S1	0.02201 (17)	0.01991 (18)	0.01649 (16)	0.00247 (12)	0.00609 (12)	−0.00049 (12)
N3	0.0184 (5)	0.0162 (5)	0.0214 (5)	0.0019 (4)	0.0056 (4)	−0.0010 (4)
C9	0.0277 (7)	0.0185 (7)	0.0348 (8)	0.0044 (6)	0.0148 (6)	−0.0034 (6)

### Geometric parameters (Å, °)

**Table:**

C1—C6	1.3943 (19)	C6—H6	0.9500
C1—C2	1.4006 (18)	C7—N1	1.2841 (18)
C1—C7	1.4642 (18)	C7—H7	0.9500
C2—C3	1.3886 (18)	N1—N2	1.3812 (15)
C2—H2	0.9500	N2—C8	1.3518 (17)
C3—O3	1.3632 (16)	N2—H2N	0.876 (9)
C3—C4	1.3952 (19)	C8—N3	1.3251 (18)
O3—H3	0.837 (9)	C8—S1	1.7038 (14)
C4—O4	1.3815 (16)	N3—C9	1.4556 (18)
C4—C5	1.3937 (19)	N3—H3N	0.879 (9)
O4—H4	0.836 (9)	C9—H9A	0.9800
C5—C6	1.3901 (19)	C9—H9B	0.9800
C5—H5	0.9500	C9—H9C	0.9800
			
C6—C1—C2	119.39 (12)	N1—C7—C1	121.28 (12)
C6—C1—C7	122.50 (12)	N1—C7—H7	119.4
C2—C1—C7	118.10 (12)	C1—C7—H7	119.4
C3—C2—C1	120.98 (12)	C7—N1—N2	115.24 (12)
C3—C2—H2	119.5	C8—N2—N1	119.45 (11)
C1—C2—H2	119.5	C8—N2—H2N	119.4 (12)
O3—C3—C2	118.57 (12)	N1—N2—H2N	120.9 (12)
O3—C3—C4	122.33 (12)	N3—C8—N2	116.78 (12)
C2—C3—C4	119.08 (12)	N3—C8—S1	124.08 (11)
C3—O3—H3	111.0 (14)	N2—C8—S1	119.13 (10)
O4—C4—C5	122.08 (12)	C8—N3—C9	124.91 (13)
O4—C4—C3	117.63 (12)	C8—N3—H3N	116.8 (12)
C5—C4—C3	120.28 (12)	C9—N3—H3N	118.1 (12)
C4—O4—H4	104.4 (14)	N3—C9—H9A	109.5
C6—C5—C4	120.34 (13)	N3—C9—H9B	109.5
C6—C5—H5	119.8	H9A—C9—H9B	109.5
C4—C5—H5	119.8	N3—C9—H9C	109.5
C5—C6—C1	119.83 (13)	H9A—C9—H9C	109.5
C5—C6—H6	120.1	H9B—C9—H9C	109.5
C1—C6—H6	120.1		

### Hydrogen-bond geometry (Å, °)

**Table:**

*D*—H···*A*	*D*—H	H···*A*	*D*···*A*	*D*—H···*A*
N3—H3N···N1	0.88 (1)	2.22 (2)	2.6282 (17)	108 (1)
O3—H3···O4^i^	0.84 (1)	2.07 (1)	2.8071 (14)	147 (2)
O4—H4···S1^ii^	0.84 (1)	2.37 (1)	3.1899 (11)	167 (2)
N2—H2N···S1^iii^	0.88 (1)	2.79 (1)	3.5766 (13)	151 (2)
C9—H9A···O4^iv^	0.98	2.56	3.435 (2)	148

Symmetry codes: (i) −*x*, −*y*, −*z*−1; (ii) *x*−1, *y*, *z*−1; (iii) −*x*+1, −*y*, −*z*+1; (iv) *x*, *y*, *z*+1.
